# A pH-responsive PEG coating strategy for enhancing the enrichment of small extracellular vesicles towards disease regions with acidic microenvironment

**DOI:** 10.1016/j.mtbio.2025.101878

**Published:** 2025-05-17

**Authors:** Jianwei Zhao, Xinyu Niu, Lei Luo, Ji Yuan, Juntao Zhang, Xin Niu, Hengli Tian, Yunlong Yang, Zhifeng Deng, Yang Wang

**Affiliations:** a Department of Neurosurgery, Shanghai Sixth People's Hospital Affiliated to Shanghai Jiao Tong University School of Medicine, No. 600 Yishan Road, Shanghai, 200233, China; b The Institute of Microsurgery on Extremities, Department of Orthopedic Surgery, Shanghai Sixth People's Hospital Affiliated to Shanghai Jiao Tong University School of Medicine, No. 600 Yishan Road, Shanghai, 200233, China; c School of Biomedical Engineering, Shanghai Jiao Tong University, No. 1954 Huashan Road, Shanghai, 200030, China

**Keywords:** pH responsive PEG coating, Engineering of sEVs, sEVs based therapy, Brain disease

## Abstract

The clinical translation of small extracellular vesicles (sEVs) as nanocarriers and therapeutic agents is severely hindered by their rapid clearance, leading to significant off-target effects. Polyethylene glycol (PEG) coating of sEVs provides a straightforward approach to address this challenge, yet it compromises their cellular internalization. To overcome this issue, we developed an acid-responsive PEG coating strategy for sEVs using 2,5-dihydroxy-4-methyl-2,5-dioxo-3-furanpropanoic acid (CDM)-modified methoxy PEG (mPEG-CDM). Western blot analysis and cellular uptake studies demonstrated that mPEG-CDM anchors to sEV membrane proteins through acid-labile cis-aconityl bonds, significantly reducing macrophage-mediated phagocytosis under physiological conditions, while restoring cellular internalization in endothelial cells (bEnd.3) and tumor cells (GL261) under weakly acidic conditions. *In vivo* imaging revealed that mPEG-CDM-modified sEVs, derived from glioma cells (GsEVs) and induced pluripotent stem cells (IsEVs), selectively accumulated in glioma tumor sites and ischemic brain regions in orthotopic glioma and stroke mouse models, respectively. Furthermore, *in vivo* studies demonstrated enhanced anti-tumor efficacy of GsEVs as drug carriers for glioma therapy and improved angiogenesis in ischemic stroke using IsEVs. Overall, this pH-responsive PEG coating strategy provides an effective approach for passive enrichment and offers valuable guidance for the design of surface-engineered sEVs in disease therapy.

## Graphical abstract

pH-responsive PEGylation strategy for the delivery of mPEG-CDM-modified sEVs, where the PEG coating stabilizes the vesicles under physiological conditions (pH ∼7.4), extending their circulation time, and is cleaved in acidic environments to promote their passive accumulation at disease sites in both glioma and ischemic stroke.

**Figure:**
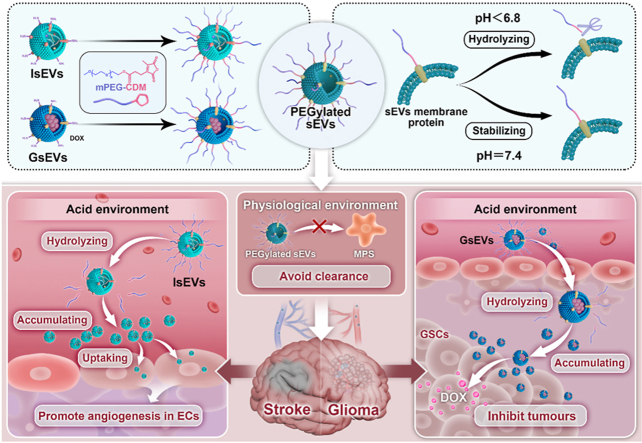

## 1 Introduction

Small extracellular vesicles (sEVs) are nanoscale, cell-derived vesicles enclosed by a phospholipid bilayer membrane that incorporates membrane proteins and parent cell-derived cargos, such as mRNA and functional proteins, to facilitate intercellular communication [1]. These vesicles offer numerous advantages, including excellent biocompatibility, diverse biofunctions determined by their cell of origin, intrinsic tropism, and the ability to cross biological barriers [[2], [3], [4]]. Furthermore, various engineering strategies have been developed to enable scalable production, efficient exogenous cargo loading, and functional surface modifications of sEVs [[5], [6], [7]]. With numerous clinical trials underway, sEVs hold great promise as next-generation drug delivery vehicles and mediators for cell-based therapies [8,9].

Regardless of their use as drug delivery carriers or therapeutic agents, sEVs are typically administered intravenously, allowing their circulation and distribution to disease sites via the bloodstream. Unfortunately, sEVs are rapidly cleared from circulation, often within just a few minutes, by the mononuclear phagocyte system (MPS) and the reticuloendothelial system (RES) [10,11]. This clearance occurs faster than that of most artificial nanoparticles and is mainly attributed to the diverse interactions arising from the complex components of sEVs [12]. As a result, rapid clearance by the MPS and RES significantly increases off-target effects and severely hinders the clinical translation of sEVs.

To overcome this limitation, several surface engineering strategies have been developed, primarily focusing on the genetic fusion of functional proteins with sEV membranes and the introduction of polymeric coatings [13,14]. Notably, functional proteins, such as albumin-binding domains, have been successfully fused to sEV membrane proteins to mitigate rapid clearance in the bloodstream [15]. However, the implementation of genetically fused membrane proteins remains complex. This approach requires the establishment of stable sEV-secreting cell lines capable of expressing the fusion proteins and effectively sorting them to the sEV membrane. Consequently, this method often results in low success rates, limited generalizability, and constrained translational value. In contrast, coating the sEV surface with synthetic polymers presents a more straightforward and flexible approach that can be applied after sEV production [10,16,17]. Polyethylene glycol (PEG) is a widely employed building block in artificial nanoparticles, providing steric stabilization and extending blood circulation time by shielding against protein opsonization and immune cell uptake [18,19]. Recent studies have introduced PEG to sEV surfaces through post-insertion or linkage to membrane proteins [10,20]. PEGylation has successfully extended the circulation time of sEVs from minutes to hours, demonstrating its effectiveness in reducing uptake by the MPS and RES [21]. However, despite its advantages in evading fast clearance, current research suggests that PEGylation does not substantially enhance sEV accumulation at disease sites, such as solid tumors. A key limitation is that PEGylation may also hinder the cellular uptake of sEVs by non-immune cells at target sites [22]. Thus, there is an urgent need for novel PEG coating strategies to improve sEV enrichment at specific pathological regions.

**Scheme 1:**
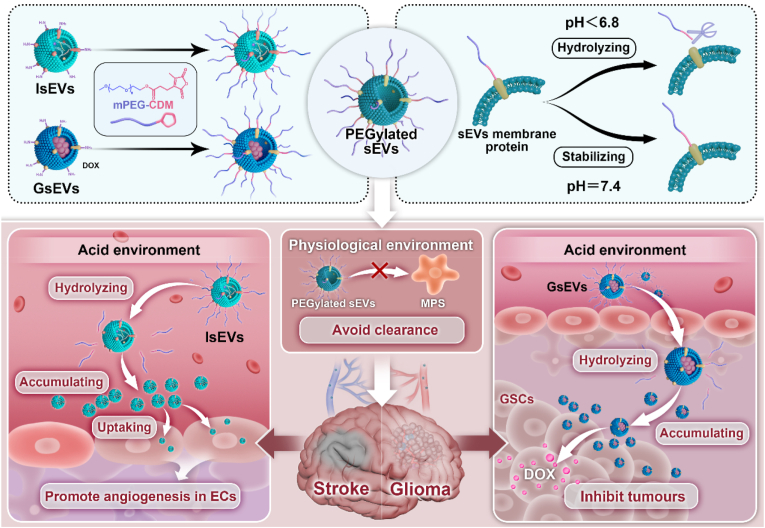
pH-responsive PEGylation strategy for the delivery of mPEG-CDM-modified sEVs, where the PEG coating stabilizes the vesicles under physiological conditions (pH ∼7.4), prolonging their circulation time, and is cleaved in acidic environments to promote their passive accumulation at disease sites in both glioma and ischemic stroke.

To address this challenge, we developed an acid-removable PEGylation strategy for sEVs. Acidic microenvironments (pH < 6.8) are common in various diseases, including tumors, inflammation, and ischemic stroke [23,24]. In glioma and ischemic stroke, extracellular pH often drops to ∼6.5 and may reach 6.0 in severely hypoxic or necrotic regions, providing a physiological basis for designing pH-responsive delivery systems [23,25]. As illustrated in Scheme 1, our approach utilizes an acid-labile cis-aconityl bond formed between free amino groups in sEV membrane proteins and methoxy PEG (mPEG) modified with CDM (2,5-dihydroxy-4-methyl-2,5-dioxo-3-furanpropanic acid), hereafter referred to as mPEG-CDM. This cis-aconityl bond is cleaved in weakly acidic environments [[26], [27], [28]]. The PEGylated sEVs generated through this method maintain a stable PEG coating under neutral pH conditions, thereby reducing clearance by MPS cells. Under mildly acidic conditions, the PEG coating is removed, facilitating enhanced uptake of sEVs by local cells. To validate the mPEG-CDM coating strategy, we employed two distinct sEV models: glioma cell-derived sEVs (GsEVs), known for their intrinsic glioma-targeting capabilities [29], and induced pluripotent stem cell (iPSC)-derived sEVs (IsEVs), which have demonstrated anti-inflammatory and pro-angiogenic effects in preclinical studies [[29], [30], [31]]. These complementary properties allowed us to investigate the coating strategy across two representative disease conditions: glioma and ischemic stroke. This study demonstrates that the acid-labile PEGylation strategy significantly enhances the accumulation of therapeutic sEVs (doxorubicin (DOX)-loaded GsEVs and IsEVs) at brain disease sites, thereby improving therapeutic efficacy for glioma and stroke, respectively. Collectively, this microenvironment-responsive PEGylation strategy provides a feasible method for passive enrichment and offers valuable insights into the design of surface-engineered sEVs for disease therapy.

## 2 Materials and methods

### 2.1 Materials

The following reagents were used: high-glucose Dulbecco's Modified Eagle Medium (DMEM) and F12 medium (Corning, USA); fetal bovine serum (FBS), phosphate-buffered saline (PBS), penicillin-streptomycin, and DAPI (Gibco, USA); HEPES buffer (Thermo Fisher Scientific, USA); vitronectin-coated plates and Nova Supplement (Nuwacell Biotechnology, China); radio-immunoprecipitation assay (RIPA) buffer, phenylmethanesulfonyl fluoride (PMSF), SDS-PAGE loading buffer, and Coomassie Brilliant Blue staining kit (Beyotime Biotechnology, China); DiD dye, DiR dye, and 4 % paraformaldehyde (Thermo Fisher Scientific, USA); CCK-8 assay kit (Dojindo, Japan); Annexin V-FITC/PI apoptosis detection kit (Beyotime Biotechnology, China); EdU cell proliferation kit (Thermo Fisher Scientific, USA); PVDF membranes (Millipore, USA); ultrafiltration centrifuge tubes (100 kDa, Millipore, USA); anti-CD9 antibodies (Abcam, ab92726, USA), anti-CD63 (Abcam, ab134045, USA), anti-ITGB1 (Santa Cruz Biotechnology, sc-374429, USA), anti-calnexin (Abcam, ab75801, USA), anti-TSG101 antibodies (Santa Cruz Biotechnology, sc-136111, USA), and anti-FLAG antibodies (Abcam, ab1162, UK); HRP-conjugated secondary antibodies (Beyotime Biotechnology, A0216, China); Matrigel (Corning, USA) and ECL kit (Bio-Rad, USA).

Key materials for chemical synthesis included 2,5-dihydroxy-4-methyl-2,5-dioxo-3-furanpropanoic acid (CDM), oxalyl chloride, N, N-dimethylformamide (DMF), dry dichloromethane (DCM), pyridine, diethyl ether, and anhydrous sodium sulfate (all from Sigma-Aldrich, USA).

Cell lines used in this study included GL261 murine glioblastoma cells, HFF-1 human lung fibroblast cells, bEnd.3 mouse brain endothelial cells, RAW 264.7 murine macrophage cells (all from the Cell Bank of the Chinese Academy of Sciences, Shanghai, China), and human induced pluripotent stem cells (iPSCs; nciPS01, RC01001-A; Nuwacell Biotechnology, Anhui, China). All cell lines were confirmed to be mycoplasma-free prior to use.

The following instruments were used: Optima XPN-100 Ultracentrifuge with SW32 Ti rotor (Beckman Coulter Life Sciences, USA); ChemiDoc MP imaging system (Bio-Rad, USA); DMI8 and DMI6 fluorescence microscopes (Leica Microsystems, Germany); nanoflow cytometer (N30 Nanoflow Analyzer, nanoFCM Inc., China); Cytoflex flow cytometer (Beckman Coulter, USA); H-7650 transmission electron microscope (Hitachi, Japan); IVIS Spectrum imaging system (PerkinElmer, USA); and Varioskan LUX multifunctional microplate reader (Thermo Fisher Scientific, USA).

All materials, reagents, and instruments were used following the manufacturer's protocols unless otherwise specified.

### 2.2 Isolation of sEVs

sEVs were isolated from GL261, iPSCs, and HFF-1 cells following the 2023 MISEV guidelines for sEV isolation and purification [32]. Cells were seeded in T75 flasks at approximately 80 % confluence and cultured in serum-free media for 48 h. The supernatant was collected and subjected to sequential centrifugation: 400×*g* for 10 min at 4 °C to remove intact cells, followed by 2000×*g* for 15 min to eliminate large debris. The resulting supernatant was further centrifuged at 10,000×*g* for 30 min at 4 °C to remove smaller particulate matter. Next, the clarified supernatant was ultracentrifuged at 100,000×*g* for 75 min at 4 °C using an Optima XPN-100 ultracentrifuge equipped with an SW32 Ti rotor. The resulting sEV pellet was washed with sterile PBS and subjected to a second ultracentrifugation under identical conditions to ensure high-purity isolation. Purified sEVs were resuspended in PBS and stored at −80 °C until further use.

### 2.3 Synthesis of mPEG-CDM

mPEG-CDM was synthesized using a previously established protocol [26]. Briefly, 0.28 g of CDM and 40 μL of DMF were dissolved in 10 mL of dry DCM. To this solution, 0.38 g of oxalyl chloride was added dropwise at 0 °C under continuous stirring. The reaction mixture was maintained at this temperature for 30 min, followed by stirring at room temperature for an additional 2 h. After completion, DCM was removed under vacuum. Next, 1 g of mPEG-NH_2_ was dissolved in 20 mL of fresh DCM and added to the residue, along with a catalytic amount of pyridine. The reaction proceeded at room temperature for 12 h.

To terminate the reaction, a saturated NH_4_Cl solution was added, and the organic layer was separated. The organic phase was washed sequentially with saturated saline solution, dried over anhydrous sodium sulfate, and concentrated using a rotary evaporator. The final residue was washed three times with diethyl ether to yield mPEG-CDM as a pale-yellow powder.

The product's structure was confirmed via UV–Vis spectroscopy and ^1^H NMR analysis, confirming the successful conjugation of CDM to mPEG.

### 2.4 Conjugation of mPEG-CDM with sEVs

Isolated sEVs from GL261, iPSCs, and HFF-1 cells were resuspended in 0.01M sodium bicarbonate buffer (pH 8.4) at a concentration of 1 × 10^10^ particles/mL to prepare for mPEG-CDM conjugation. To form mPEG-CDM-sEV conjugates, mPEG-CDM was dissolved in the same buffer at a final concentration of 4 mg/mL. Equal volumes of the sEV suspension and the mPEG-CDM solution were combined and incubated at 25 °C for 2 h under gentle stirring, allowing the formation of pH-responsive cis-aconityl bonds between mPEG-CDM and sEV membrane proteins. Post-reaction, the modified sEVs were purified by ultracentrifugation at 100,000×*g* for 75 min at 4 °C to remove unreacted mPEG-CDM. The pellet was washed with sterile PBS, followed by a second ultracentrifugation under identical conditions to ensure high purity. The final mPEG-CDM-sEV conjugates were resuspended in sterile PBS and stored at −80 °C for subsequent analyses.

### 2.5 Coomassie Brilliant Blue staining

To evaluate the binding ability of mPEG-CDM, BSA was used as a model protein. BSA was dissolved in 0.01 M sodium bicarbonate buffer (pH 8.4) at a concentration of 2 mg/mL mPEG-CDM solutions were prepared at concentrations of 1, 2, 4, and 8 mg/mL in the same buffer. Equal volumes of the BSA solution and mPEG-CDM solutions were mixed and incubated at 25 °C for 2 h under gentle stirring to allow conjugation. After the reaction, the samples were mixed with 5 × SDS-PAGE loading buffer and boiled at 95 °C for 10 min. SDS-PAGE was performed to separate the proteins, and the bands were visualized using Coomassie Brilliant Blue staining.

For the acid responsiveness test, BSA modified with 4 mg/mL mPEG-CDM was transferred into neutral (0.01M PBS, pH 7.4) or acidic (0.01 M sodium acetate buffer, pH 6.0) conditions and incubated at 25 °C for 30 min. After incubation, the samples were mixed with SDS-PAGE loading buffer, boiled at 95 °C, and analyzed by SDS-PAGE. Protein bands were visualized with Coomassie Brilliant Blue staining to assess the pH-dependent behavior of mPEG-CDM.

### 2.6 Western blot and pH behavior analysis

sEVs derived from GL261, iPSCs, and HFF-1 cells were lysed using RIPA buffer supplemented with PMSF, and protein concentrations were quantified using a BCA assay to ensure equal protein loading. The samples were mixed with 5 × SDS-PAGE loading buffer, boiled at 95 °C for 10 min, and separated by SDS-PAGE. The resolved proteins were transferred onto PVDF membranes under standard transfer conditions.

For Western blot analysis, membranes were blocked with 5 % non-fat milk in TBST for 1 h at room temperature, followed by overnight incubation at 4 °C with primary antibodies: anti-CD9 to detect surface proteins and anti-TSG101 to verify internal protein integrity. After three washes with TBST, membranes were incubated with HRP-conjugated secondary antibodies for 1 h at room temperature. Protein bands were visualized using an ECL kit and imaged with the ChemiDoc MP system.

For pH-dependent behavior analysis, mPEG-CDM-sEV conjugates were incubated in PBS (pH 7.4) or sodium acetate buffer (pH6.5 or pH 6.0) for 30 min at room temperature. After incubation, samples were analyzed by SDS-PAGE and Western blot to assess changes in conjugation and protein binding under different pH conditions.

### 2.7 Cellular uptake assay

RAW 264.7, GL261, and bEnd.3 cells were seeded into 24-well plates at a density of 1 × 10^5^ cells per well and allowed to adhere overnight at 37 °C in a humidified 5 % CO_2_ atmosphere. DiD-labeled sEVs or mPEG-CDM-modified sEVs (1 × 10^9^ particles/mL) were added and incubated for specified time periods (2, 6, or 12 h). After incubation, cells were washed three times with PBS to remove unbound sEVs, fixed with 4 % paraformaldehyde for 20 min, and stained with DAPI to visualize nuclei. Cellular uptake was observed by fluorescence microscopy, and quantitative analysis of fluorescence intensity was performed using flow cytometry.

To simulate an acidic environment, mPEG-CDM-modified sEVs were adjusted to pH 6.0 using 1 mM HCl and incubated at room temperature for 10 min. The modified sEVs were added to RAW 264.7 cells and incubated for 12 h. Cells were then washed, fixed, stained, and analyzed by microscopy and flow cytometry as described above.

For pH-dependent uptake studies in GL261 and bEnd.3 cells, high-glucose DMEM adjusted to pH 7.4, 6.5, or 6.0 with 25 mM HEPES buffer was used. DiD-labeled mPEG-CDM-GsEVs or mPEG-CDM-IsEVs (1 × 10^9^ particles/mL) were added and incubated at 37 °C in a 5 % CO_2_ atmosphere for the specified time periods. Cells were processed as described above for microscopy and flow cytometry to evaluate uptake efficiency under different pH conditions.

### 2.8 Animal models for glioma and stroke

All animal experiments were approved by the Animal Research Committee of Shanghai Sixth People's Hospital (DWSY2022-0176) and conducted in accordance with the Guide for the Care and Use of Laboratory Animals published by the US National Institutes of Health (NIH publication, 8th edition, 2011). Male nude mice (2–3 months old, 18–25 g) and male C57BL/6 mice (2–3 months old, 25–30 g) were housed under standard conditions with a 12-h light/dark cycle and allowed ad libitum access to food and water.

For the glioma model, GL261 cells (1 × 10^6^ in 5 μL sterile PBS) were stereotactically injected into the right striatum using a NeuroStar stereotactic instrument and a microinjection pump for precise delivery. Mice were monitored daily for health status and tumor development over three weeks.

For the MCAO stroke model, male C57BL/6 mice were anesthetized with 5 % isoflurane for induction and maintained with 2 % isoflurane during surgery. A silicon-coated suture (Doccol, USA) was inserted through the external carotid artery and advanced into the middle cerebral artery to induce occlusion for 60 min, followed by reperfusion upon suture removal. Regional cerebral blood flow (rCBF) was continuously monitored, and only mice with an rCBF reduction >50 % were included. Animals with post-MCAO complications or mortality were excluded from subsequent experiments.

### 2.9 sEVs distribution analysis

Mice received 200 μL of labeled sEVs (2 × 10^11^ particles/mL) via tail vein injection. In glioma-bearing nude mice, DiR-labeled sEVs derived from GL261 cells were administered on day 21 post-tumor implantation. For the MCAO stroke model, DiR-labeled sEVs and CD9-mCherry-FLAG sEVs derived from iPSCs were injected immediately after reperfusion.

IVIS imaging was conducted at specific time points to evaluate sEV biodistribution. For glioma models, imaging was performed at 2, 12, and 24 h post-injection, while for stroke models, imaging was carried out at 2 and 12 h post-injection. Mice were anesthetized with 2 % isoflurane during imaging to ensure consistent signal acquisition. Afterward, mice were euthanized, and organs, including the brain, liver, heart, lungs, spleen, kidneys, and intestines, were collected for *ex vivo* fluorescence analysis.

Brain and liver tissue sections were fixed in 4 % paraformaldehyde, dehydrated in a sucrose gradient, and sectioned at 10 μm thickness. Fluorescence signals from DiR-labeled sEVs were analyzed using a Leica DMI6 fluorescence microscope. For the MCAO model, mCherry fluorescence from CD9-mCherry-FLAG sEVs was also assessed to confirm localization. Immunohistochemical staining with anti-FLAG antibodies was performed to further validate the localization of FLAG-tagged sEVs.

### 2.10 DOX loading

To prepare DOX-loaded mPEG-CDM-modified sEVs, 1 × 10^10^ mPEG-CDM-modified sEVs were suspended in PBS containing 0.2 % (w/v) saponin and incubated with 100 μM DOX at room temperature for 1 h to facilitate drug loading. As previously described [33], this protocol allows simultaneous drug loading and content removal. After incubation, the solution was washed and concentrated using a 100 kDa ultrafiltration centrifuge tube to remove unbound DOX. This washing process was repeated five times with PBS to ensure complete removal of free DOX. The final DOX-loaded sEVs were resuspended in sterile PBS for downstream experiments.

The DOX concentration in sEVs was quantified by measuring fluorescence intensity (excitation: 480 nm; emission: 595 nm) using a multifunctional microplate reader. A pre-established standard curve (y = 1.73573x − 0.679177, R^2^ = 0.993) was used to calculate the concentration, where y represents the DOX concentration and x represents the measured fluorescence signal. DOX loading efficiency (%) was calculated using the formula: DOX Loading Efficiency (%) = (Amount of DOX in sEVs/Total DOX) × 100 %.

### 2.11 Statistical analysis

All statistical analyses were performed using GraphPad Prism (version 8.2.1). Data are expressed as mean ± standard deviation (SD). Comparisons between two groups were analyzed using an unpaired Student's t-test. For comparisons among three or more groups, one-way ANOVA was applied for data following a normal distribution with equal SDs. Nonparametric tests were used for non-normally distributed data, while the Brown-Forsythe and Welch ANOVA tests were applied for data with unequal SDs. For repeated measures over time across multiple groups, two-way repeated measures ANOVA was performed. Survival analyses were conducted using the Kaplan-Meier method, and differences between survival curves were assessed with the log-rank test.

Statistical significance was set at p < 0.05 and denoted as follows: ns (not significant), ∗p < 0.05, ∗∗p < 0.01, and ∗∗∗p < 0.001. All statistical tests and visualizations were standardized and executed in GraphPad Prism to ensure consistency and accuracy.

## 3 Results and discussion

### 3.1 Construction and characterization of mPEG-CDM sEVs

In this study, the cis-aconityl bond, formed between CDM and free amino groups, was employed as a chemical tool to achieve the pH-responsive PEG coating of sEVs. Extensively utilized in the construction of artificial acid-responsive drug carriers and polymer-drug conjugates, the cis-aconityl bond is well-regarded for its structural simplicity and precise responsiveness to mildly acidic environments [26,34]. Moreover, the abundant membrane proteins present on sEVs provide ample free amino groups, making them highly suitable for PEG coating via cis-aconityl linkage. To implement this strategy, mPEG-CDM (10 kDa) was synthesized as previously described (Fig. 1a) [26]. The successful conjugation of CDM to mPEG was confirmed by the UV–Vis spectroscopy, which exhibited a similar absorbance profile to CDM alone (Fig. 1b). Furthermore, the ^1^H NMR spectrum identified characteristic peaks corresponding to the CDM moiety, indicating a modification efficiency over 90 % (Fig. S1).

**Fig. 1:**
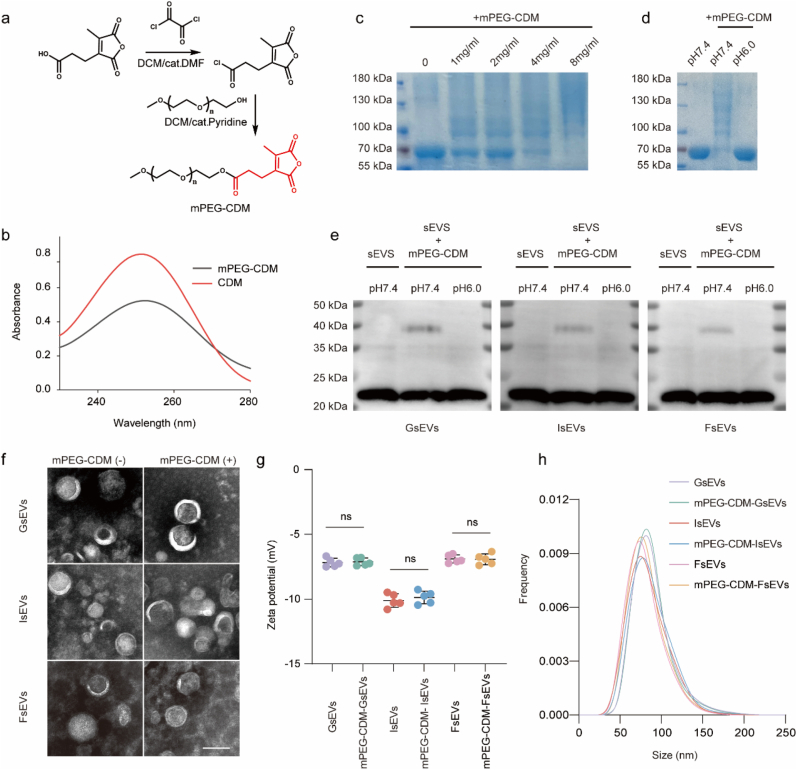
Impact of mPEG-CDM modification on sEV properties. (a) Chemical structure of mPEG-CDM. (b) UV–Vis spectra of CDM and mPEG-CDM. (c) Coomassie Brilliant Blue staining of BSA modified with mPEG-CDM at concentrations of 0, 1, 2, 4, and 8 mg/mL. (d) Coomassie Brilliant Blue staining of BSA alone and BSA modified with mPEG-CDM under pH 7.4 and pH 6.0 conditions. (e) Western blot analysis of CD9 in GsEVs, IsEVs, and FsEVs before and after mPEG-CDM modification under pH 7.4 and pH 6.0 conditions. (f) Representative TEM images of GsEVs, IsEVs, and FsEVs before and after mPEG-CDM modification. Scale bar: 100 nm. (g) Zeta potential of GsEVs, IsEVs, and FsEVs before and after mPEG-CDM modification. (h) Particle size distribution of GsEVs, IsEVs, and FsEVs before and after mPEG-CDM modification. Data are shown as mean ± SD (n = 5). ns, not significant. (For interpretation of the references to colour in this figure legend, the reader is referred to the Web version of this article.)

To investigate the protein conjugation ability of mPEG-CDM and evaluate its acid-responsiveness, bovine serum albumin (BSA) was selected as a model protein. When BSA was incubated with varying concentrations of mPEG-CDM, Coomassie Brilliant Blue staining demonstrated a clear, concentration-dependent upward shift in the BSA band, indicating successful mPEG-CDM binding (Fig. 1c). Upon transferring the mPEG-CDM-modified BSA to buffers with either neutral (pH 7.4) or acidic (pH 6.0) conditions, the modified BSA retained an upward-shifted band under neutral pH, while the acidic buffer restored the migration pattern to that of unmodified BSA. This observation confirmed the stability of PEG modification under physiological conditions and its cleavage under acidic environments (Fig. 1d).

We applied the mPEG-CDM coating strategy to sEVs from GL261 glioma cells (GsEVs), iPSCs (IsEVs), and HFF-1 human foreskin fibroblasts (FsEVs) to validate its versatility across diverse sEV sources. The iPSCs used for IsEVs were identified through specific markers to ensure consistency with the cell type used in downstream experiments (Fig. S2). Western blot analysis further confirmed that CD9, CD63, and ITGB1 were enriched in all sEV groups, while Calnexin, a negative marker for cellular contamination, was absent, indicating high sample purity (Fig. S3). Unlike synthetic nanoparticles, the abundant membrane proteins on sEVs provide a rich repertoire of binding sites for mPEG-CDM attachment. To evaluate the coating efficiency, CD9—a well-characterized membrane protein marker of sEVs with a molecular weight of ∼24 kDa—was selected as a representative target. Isolated sEVs from all three sources were incubated with 2 mg/mL mPEG-CDM in NaHCO_3_ buffer (0.01 M), followed by buffer exchange to PBS (pH 7.4) or acidic buffer (pH 6.0) via ultracentrifugation. Western blot analysis of CD9 revealed the emergence of new bands between 30 and 40 kDa under neutral pH, indicating successful PEG conjugation to sEV membrane proteins (Fig. 1e). In addition, the successful conjugation of mPEG-CDM was also confirmed by ^1^H NMR. As shown in Fig. S4, strong peaks (δ3.5∼δ3.8) corresponding to the H in mPEG-CDM were found in all the three sEVs groups, demonstrating a high conjugation efficacy of mPEG-CDM. To simulate the moderately to severely acidic microenvironments commonly observed in glioma and ischemic stroke, we selected pH 6.5 and pH 6.0 for evaluating PEG detachment. At pH 6.0, the CD9 band nearly returned to its native position, confirming effective PEG cleavage (Fig. 1e). A similar trend was observed at pH 6.5, where the band was largely restored, indicating that the PEG coating also responds efficiently under milder acidic conditions (Fig. S5). In addition, Western blot analysis of acid-treated mPEG-CDM-sEVs showed that surface membrane proteins including CD9, CD63, and ITGB1 remained clearly detectable in all EV groups, indicating that PEG removal did not compromise surface protein integrity (Fig. S6).

The morphological integrity of sEVs post-modification was examined using transmission electron microscopy (TEM). As shown in Fig. 1f, the typical round, cup-shaped morphology of GsEVs and IsEVs was well-preserved following mPEG-CDM coating, confirming that the modification process did not compromise vesicle structure. This observation was further supported by zeta potential measurements, which showed no significant change in surface charge after mPEG-CDM modification (Fig. 1g). Consistent with these findings, nano-flow cytometry revealed negligible differences in particle size distribution between unmodified and mPEG-CDM-modified sEVs, ruling out aggregation or degradation effects during the coating process (Fig. 1h). Additionally, Western blot analysis of the internal exosomal marker TSG101 showed no detectable changes in band intensity or position, indicating that the intrinsic cargo of sEVs remained unaffected by the surface modification (Fig. S7). Furthermore, TEM revealed that mPEG-CDM-sEVs retained their typical morphology after acid-triggered PEG cleavage (pH 6.0, 30 min), with no evidence of vesicle rupture or aggregation (Fig. S8). These results underscore the effectiveness of mPEG-CDM coating as a robust strategy for enabling pH-responsive functionalization, all while maintaining the structural and biological integrity of sEVs.

### 3.2 Selective cellular uptake of sEVs enabled by pH-Responsive mPEG-CDM coating

The interaction of sEVs with macrophages and targeted cells is a key determinant of clearance rates and therapeutic efficacy. While conventional PEG coatings reduce macrophage uptake and clearance via non-cleavable bonds, these coatings often hinder target cell uptake, thereby limiting therapeutic potential. To address this limitation, the mPEG-CDM coating was designed to achieve selective uptake by reducing macrophage clearance at physiological pH (7.4) and enhancing cellular internalization under acidic conditions (pH < 6.8).

The cellular uptake behavior of mPEG-CDM-modified sEVs (mPEG-CDM-sEVs) was evaluated using DiD-labeled sEVs in three cell types: RAW264.7 macrophages (to model MPS clearance), GL261 glioma cells (tumor microenvironment), and bEnd.3 brain endothelial cells (vascular microenvironment). At pH 7.4, fluorescence microscopy revealed significantly reduced uptake of mPEG-CDM-modified GsEVs, IsEVs, and FsEVs by RAW264.7 macrophages compared to unmodified sEVs (Fig. 2a). Flow cytometry analysis further confirmed this reduction, showing significantly lower fluorescence intensity for mPEG-CDM-sEVs and highlighting the effectiveness of the mPEG-CDM coating in minimizing macrophage-mediated clearance under neutral conditions (Fig. 2b). Control experiments verified the specificity of the fluorescence signals, ruling out contributions from free DiD or unreacted materials (Fig. S9a).

**Fig. 2:**
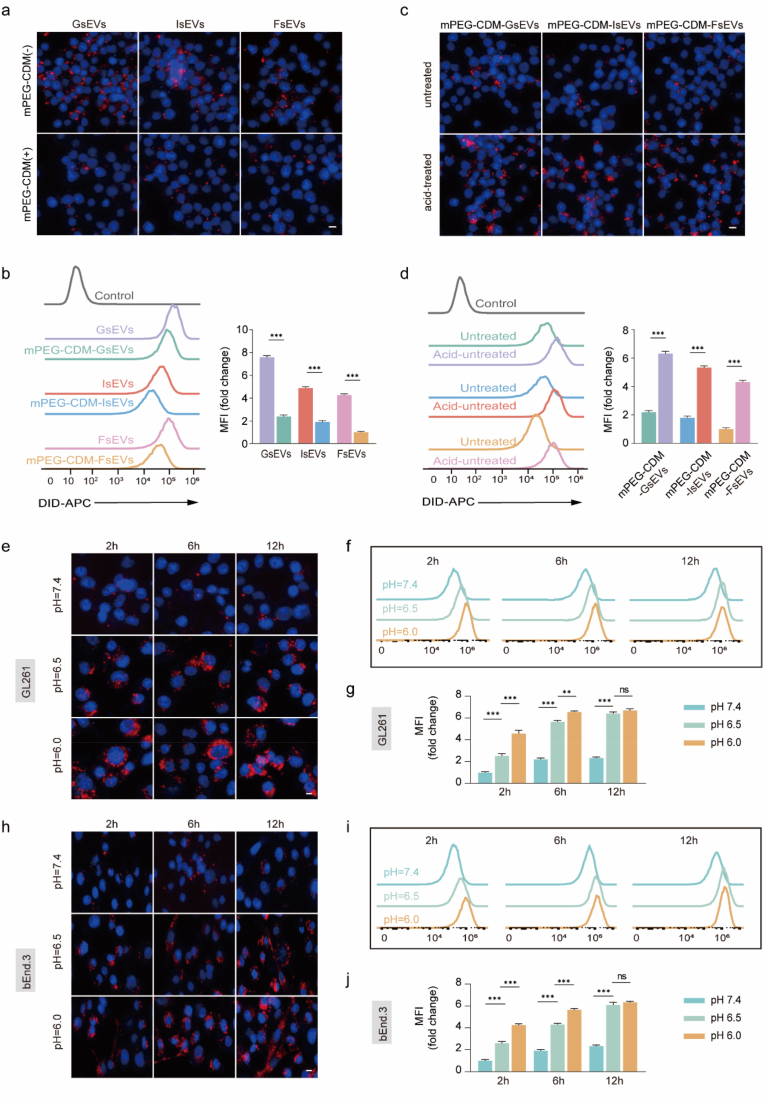
Cellular uptake and pH-responsive behavior of mPEG-CDM-modified sEVs. (a) Representative fluorescence images of RAW 264.7 cells with unmodified and mPEG-CDM-modified GsEVs, IsEVs, and FsEVs at pH 7.4. (b) Flow cytometry histograms and corresponding statistical analysis of MFI for RAW 264.7 cells with unmodified or mPEG-CDM-modified sEVs at pH 7.4. (c) Representative fluorescence images of RAW 264.7 cells with acid-pretreated and untreated mPEG-CDM-modified GsEVs, IsEVs, and FsEVs. (d) Flow cytometry histograms and corresponding statistical analysis of MFI for RAW 264.7 cells with acid-pretreated and untreated mPEG-CDM-modified sEVs. (e) Representative fluorescence images of GL261 cells with mPEG-CDM-GsEVs at pH 7.4, 6.5, and 6.0 for 2, 6, and 12 h. (f) Flow cytometry histograms of GL261 cells with mPEG-CDM-GsEVs at pH 7.4, 6.5, and 6.0 for 2, 6, and 12 h. (g) Statistical analysis of MFI from (f). (h) Representative fluorescence images of bEnd.3 cells with mPEG-CDM-IsEVs at pH 7.4, 6.5, and 6.0 for 2, 6, and 12 h. (i) Flow cytometry histograms of bEnd.3 cells with mPEG-CDM-IsEVs at pH 7.4, 6.5, and 6.0 for 2, 6, and 12 h. (j) Statistical analysis of MFI from (i). Data are shown as mean ± SD (n = 5). ∗∗p < 0.01; ∗∗∗p < 0.001; ns, not significant. DiD (red)-labeled sEVs; DAPI (blue)-labeled nuclei. Scale bar: 10 μm. (For interpretation of the references to colour in this figure legend, the reader is referred to the Web version of this article.)

To assess the pH-responsiveness of the mPEG-CDM coating, sEVs were pretreated in an acidic buffer (pH 6.0) prior to incubation with RAW264.7 macrophages. Fluorescence microscopy showed a pronounced increase in macrophage uptake of acid-treated mPEG-CDM-sEVs compared to untreated controls (Fig. 2c). This finding was further supported by flow cytometry and statistical analysis, demonstrating that the acidic environment triggered PEG detachment, thereby promoting cellular internalization (Fig. 2d).

The selective uptake of mPEG-CDM-coated sEVs was further explored in GL261 glioma cells and bEnd.3 brain endothelial cells to mimic disease-relevant microenvironments. In both cell types, fluorescence microscopy revealed a marked increase in uptake of mPEG-CDM-GsEVs (GL261, Fig. 2e) and mPEG-CDM-IsEVs (bEnd.3, Fig. 2h) under acidic conditions, with the highest uptake observed at pH 6.0. Even at pH 6.5, the uptake was significantly greater than at pH 7.4, highlighting the coating's sensitivity to mildly acidic environments. Flow cytometry confirmed this trend, with significantly higher mean fluorescence intensity (MFI) observed at pH 6.0 and 6.5 compared to pH 7.4 in both GL261 (Fig. 2f and g) and bEnd.3 cells (Fig. 2i and j). In contrast, unmodified sEVs showed no significant differences in uptake across pH conditions (Fig. S9b), confirming that the enhanced internalization of mPEG-CDM-sEVs was driven by acid-sensitive PEG detachment. This pH-responsive mechanism offers a dual benefit: minimizing macrophage-mediated clearance at physiological pH while promoting efficient target cell uptake in acidic microenvironments. This unique property enhances the therapeutic potential of mPEG-CDM-sEVs by improving delivery to disease sites while minimizing off-target clearance.

### 3.3 Improved *in vivo* enrichment of mPEG-CDM-modified sEVs at disease sites

The *in vivo* enrichment of mPEG-CDM-modified sEVs in glioma and ischemic stroke regions was investigated to assess how their pH-responsive properties influence localization. Both glioma and ischemic stroke microenvironments are characterized by acidity due to pathological mechanisms such as hypoxia-induced metabolic shifts and the accumulation of acidic byproducts. In glioma, acidity results from the Warburg effect, where glioma cells rely on anaerobic glycolysis to produce lactic acid, as well as from impaired proton export, rapid tumor growth, and abnormal vascularization, all of which contribute to the acidic microenvironment [[35], [36], [37]]. Similarly, ischemic stroke induces brain acidosis due to hypoxia-driven anaerobic metabolism and mitochondrial dysfunction, creating an environment conducive to pH-responsive strategies [23,25,38]. In addition to acidity, blood-brain barrier disruption in both glioma and ischemic stroke further enhances sEV accumulation by increasing vascular permeability, which facilitates passive penetration into brain lesions [30,33].

To evaluate sEV enrichment in glioma, DiR-labeled GsEVs and mPEG-CDM-GsEVs were intravenously injected into GL261 glioma orthotopic models, leveraging the intrinsic homing ability of GsEVs to glioma cells. Biodistribution was monitored at 2, 12, and 24 h post-injection using IVIS imaging. In both glioma and liver regions, fluorescence signals gradually increased, peaking at 12 h before declining at 24 h, indicating a time-dependent enrichment profile. At all-time points, mPEG-CDM-GsEVs exhibited significantly stronger fluorescence signals in glioma regions and lower hepatic accumulation compared to unmodified GsEVs, confirming the role of PEGylation in reducing MPS clearance and enhancing tumor enrichment (Fig. 3a and b). *Ex vivo* IVIS imaging of major organs corroborated these findings, showing enhanced tumor fluorescence along with reduced liver localization for mPEG-CDM-GsEVs compared to unmodified GsEVs (Fig. 3c and d). Fluorescence microscopy of brain and liver sections further validated these results, revealing higher fluorescence signals in tumor regions for mPEG-CDM-GsEVs and lower off-target signals in liver sections (Fig. S10a and b). These results highlight the ability of mPEG-CDM to prolong sEV circulation, reduce systemic clearance, and enhance passive tumor enrichment through reduced MPS uptake and increased vascular permeability.

**Fig. 3:**
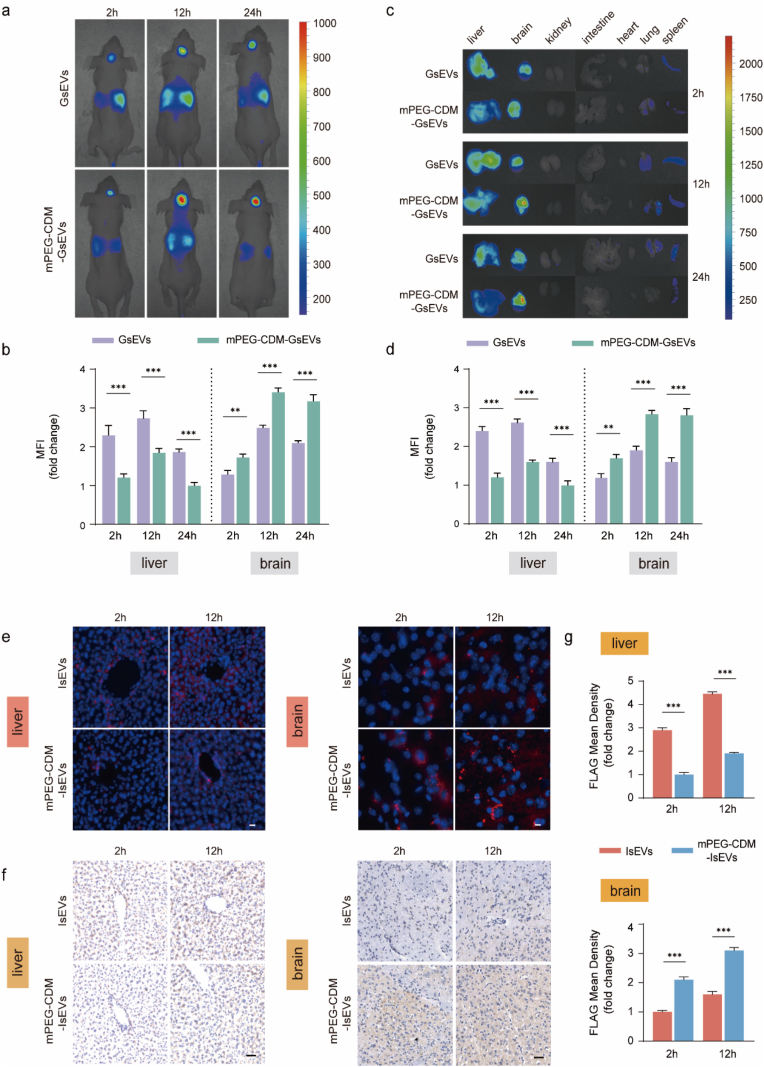
*In vivo* biodistribution of mPEG-CDM-modified sEVs. (a) IVIS fluorescence images of glioma-bearing mice injected with DiR-labeled GsEVs or mPEG-CDM-GsEVs at 2, 12, and 24 h post-injection. (b) Statistical analysis of MFI in liver and brain regions from (a). (c) *Ex vivo* fluorescence images of major organs (liver, brain, kidney, intestine, heart, lung, and spleen) at 2, 12, and 24 h post-injection. (d) Statistical analysis of MFI in liver and brain regions from (c). (e) Representative fluorescence images of liver and brain sections from MCAO mice injected with mCherry-FLAG-labeled IsEVs or mPEG-CDM-IsEVs at 2 and 12 h post-injection. Brain sections were obtained from infarct regions within the ischemic hemisphere. mCherry (red)-labeled sEVs; DAPI (blue)-stained nuclei. Scale bar: 10 μm. (f) Representative immunohistochemical images of liver and brain sections from MCAO mice injected with mCherry-FLAG-labeled IsEVs or mPEG-CDM-IsEVs at 2 and 12 h post-injection. Brain sections were obtained from infarct regions. Scale bar: 50 μm. (g) Statistical analysis of FLAG mean density in liver and brain sections from (f). Data are shown as mean ± SD (n = 5). ∗∗p < 0.01; ∗∗∗p < 0.001. (For interpretation of the references to colour in this figure legend, the reader is referred to the Web version of this article.)

For ischemic brain enrichment, both DiR- and mCherry-FLAG-labeled mPEG-CDM-IsEVs were employed in the middle cerebral artery occlusion (MCAO) stroke model to reliably assess biodistribution and cellular localization. IVIS imaging at 2 and 12 h post-injection demonstrated significantly higher DiR fluorescence signals in ischemic brain regions for mPEG-CDM-IsEVs compared to unmodified IsEVs, which showed predominant liver accumulation, whereas the observation period in the ischemic stroke model was limited to 12 h due to its relatively short duration of the acidic microenvironment (Fig. S10c and d). *Ex vivo* imaging further confirmed enhanced localization in ischemic brain regions and reduced liver accumulation for mPEG-CDM-IsEVs relative to unmodified IsEVs (Fig. S10e and f). Fluorescence microscopy of brain sections using mCherry-FLAG-labeled sEVs revealed significantly stronger mCherry signals in ischemic regions for mPEG-CDM-IsEVs, with minimal fluorescence observed in liver sections (Fig. 3e). Immunohistochemical staining of FLAG validated these observations, showing higher densities of mPEG-CDM-IsEVs in ischemic brain regions compared to unmodified IsEVs, alongside reduced liver localization (Fig. 3f and g).

The results from both GL261 glioma orthotopic and MCAO stroke models collectively demonstrate that mPEG-CDM modification significantly enhances sEV accumulation in acidic disease microenvironments, such as gliomas and ischemic brain regions. These findings highlight the potential of mPEG-CDM-modified sEVs to enhance delivery precision while minimizing systemic off-target accumulation, offering a promising strategy for enriching sEVs at acidic pathological sites.

### 3.4 Selective drug delivery by DOX-loaded mPEG-CDM-sEVs *in vitro*

The pH-responsive drug delivery properties of DOX-loaded mPEG-CDM-sEVs (mPEG-CDM-GsEVs-DOX) were evaluated using a saponin-based strategy for DOX encapsulation, ensuring efficient drug loading and retention within the sEVs [33]. The drug loading content of mPEG-CDM-GsEVs-DOX was approximately 1.15 μg/10^10^ particles (∼2 nmol/10^10^ particles). Cellular uptake efficiency was assessed under neutral (pH 7.4) and acidic (pH 6.0) conditions to simulate physiological and tumor microenvironments. Fluorescence microscopy and quantitative analysis revealed that at pH 7.4, unmodified GsEVs-DOX exhibited higher cellular uptake compared to free DOX, demonstrating the enhanced delivery efficiency of sEV-based carriers. In contrast, mPEG-CDM-GsEVs-DOX showed significantly reduced uptake at pH 7.4 due to the steric shielding effect of the PEG coating (Fig. 4a and b). However, under acidic conditions (pH 6.0), the cellular uptake of mPEG-CDM-GsEVs-DOX notably increased, demonstrating that PEG detachment facilitates DOX release and internalization. These results confirm the pH-responsive nature of mPEG-CDM-GsEVs-DOX, enabling selective drug delivery in acidic tumor environments while minimizing non-specific uptake under neutral physiological conditions. To evaluate cytotoxicity, Annexin V/PI staining and flow cytometry were used to assess apoptosis in HFF-1 fibroblasts and GL261 glioma cells. At pH 7.4, mPEG-CDM-GsEVs-DOX induced significantly lower apoptosis rates in HFF-1 cells compared to both free DOX and unmodified GsEVs-DOX, highlighting the protective effect of the PEG coating in neutral environments (Fig. 4c and d). Conversely, at pH 6.0, mPEG-CDM-GsEVs-DOX induced apoptosis rates in GL261 cells similar to those induced by unmodified GsEVs-DOX, reflecting the reactivation of cytotoxicity through PEG detachment and efficient DOX delivery. Notably, free DOX induced lower apoptosis rates in both cell types compared to sEV-encapsulated formulations, emphasizing the superior therapeutic efficacy and tumor-targeting capability of sEV-based drug delivery systems.

**Fig. 4:**
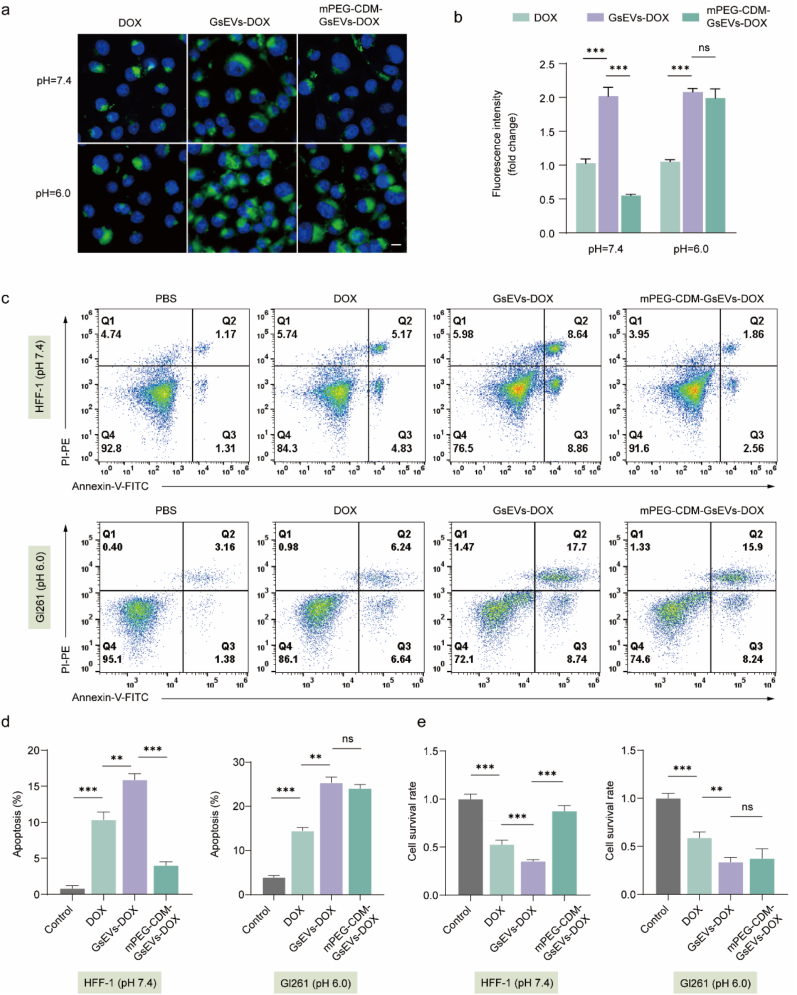
pH-dependent uptake and cytotoxicity of mPEG-CDM-GsEVs-DOX *in vitro*. (a) Representative fluorescence images of GL261 cells treated with free DOX, GsEVs-DOX, and mPEG-CDM-GsEVs-DOX under pH 7.4 and pH 6.0 conditions. DOX (green); DAPI (blue). Scale bar: 10 μm. (b) Statistical analysis of DOX fluorescence intensity from (a). (c) Representative flow cytometry scatter plots of Annexin V-FITC and PI staining for apoptosis in HFF-1 cells (pH 7.4) and GL261 cells (pH 6.0) treated with control, free DOX, GsEVs-DOX, and mPEG-CDM-GsEVs-DOX for 24 h. (d) Statistical analysis of apoptosis rates from (c). (e) Statistical analysis of cell survival rates evaluated by CCK-8 assay in HFF-1 cells (pH 7.4) and GL261 cells (pH 6.0) treated with control, free DOX, GsEVs-DOX, and mPEG-CDM-GsEVs-DOX. Data are shown as mean ± SD (n = 5). ∗∗p < 0.01; ∗∗∗p < 0.001; ns, not significant. (For interpretation of the references to colour in this figure legend, the reader is referred to the Web version of this article.)

These findings were further supported by CCK-8 cell viability assays, which showed a clear pH-dependent trend. At pH 7.4, mPEG-CDM-GsEVs-DOX preserved significantly higher viability in HFF-1 cells compared to free DOX and unmodified GsEVs-DOX, reaffirming the protective role of the PEG coating (Fig. 4e). Under acidic conditions (pH 6.0), the cell viability of GL261 glioma cells in the mPEG-CDM-GsEVs-DOX group decreased markedly, consistent with the reactivation of cytotoxicity and efficient drug delivery in the acidic tumor microenvironment.

In summary, mPEG-CDM-GsEVs-DOX demonstrates dual functionality by minimizing off-target toxicity in neutral environments and reactivating cytotoxicity under acidic conditions. By integrating PEG detachment with tumor-specific pH responsiveness, this nanocarrier system provides precise drug delivery and enhanced therapeutic specificity, establishing its potential as an adaptable platform for tumor-targeted therapy in glioma cells.

### 3.5 Impact of mPEG-CDM-IsEVs on angiogenesis in bEnd.3 cells under acidic conditions

The potential of mPEG-CDM-IsEVs to promote angiogenesis was evaluated under ischemic stroke-relevant conditions, focusing on their effects on endothelial cell migration and proliferation at neutral (pH 7.4) and acidic (pH 6.0) conditions. Endothelial cells, such as bEnd.3, play a critical role in vascular repair and angiogenesis, making them an ideal model for assessing the pro-angiogenic potential of mPEG-CDM-IsEVs in acidic environments that mimic ischemic stroke. In wound healing assays (Fig. 5a and b), unmodified IsEVs significantly enhanced bEnd.3 cell migration at pH 7.4, with the most pronounced effect observed at 12 h. In contrast, mPEG-CDM-IsEVs showed markedly reduced migration at pH 7.4, likely due to the PEG coating's inhibitory effect on cellular uptake, thereby minimizing off-target activity under non-pathological conditions. At pH 6.0, the detachment of the PEG coating restored the pro-migratory activity of mPEG-CDM-IsEVs, achieving migration levels comparable to unmodified IsEVs. Quantitative analysis of the migration area confirmed these observations, demonstrating significantly reduced migration for mPEG-CDM-IsEVs at pH 7.4 but no significant difference compared to unmodified IsEVs at pH 6.0. The transwell migration assay provided additional evidence of this pH-responsive behavior (Fig. 5c and d). At pH 7.4, bEnd.3 cells treated with mPEG-CDM-IsEVs exhibited significantly reduced migration compared to unmodified IsEVs, as shown in representative images and corresponding quantification. However, at pH 6.0, the PEG detachment reactivated the pro-migratory effects of IsEVs, leading to migration levels similar to unmodified IsEVs. To assess endothelial cell proliferation, EdU incorporation assays were conducted (Fig. 5e and f). At pH 7.4, mPEG-CDM-IsEVs exhibited significantly fewer EdU-positive cells than unmodified IsEVs, emphasizing the PEG coating's suppressive role in neutral environments. Conversely, at pH 6.0, mPEG-CDM-IsEVs significantly enhanced EdU-positive cells, matching the levels observed with unmodified IsEVs, demonstrating the restoration of pro-proliferative potential under acidic conditions.

**Fig. 5:**
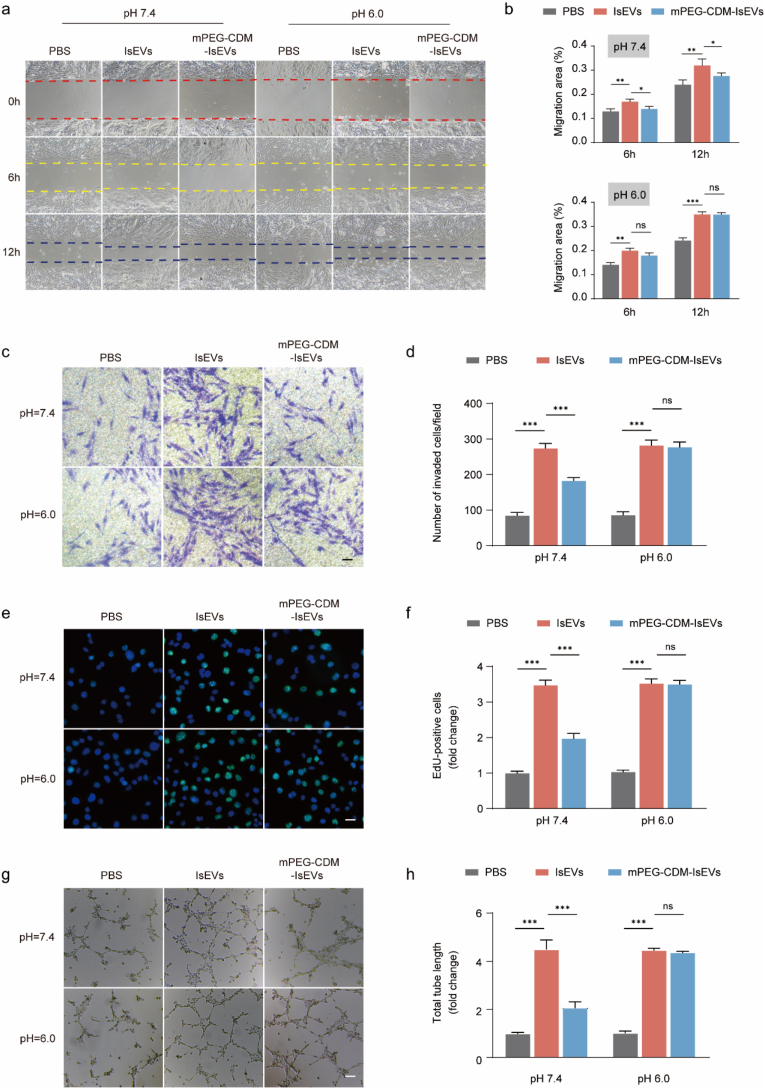
pH-dependent effects of mPEG-CDM-IsEVs on bEnd.3 migration and proliferation. (a) Representative images from wound healing assays showing bEnd.3 cell migration treated with PBS, IsEVs, or mPEG-CDM-IsEVs under pH 7.4 and pH 6.0 conditions. (b) Statistical analysis of migration area from (a). (c) Representative images from transwell migration assays showing bEnd.3 cell migration under pH 7.4 and pH 6.0 after treatment with PBS, IsEVs, or mPEG-CDM-IsEVs. Scale bar: 50 μm. (d) Statistical analysis of migrated cells from (c). (e) Representative EdU staining images of bEnd.3 cells treated with PBS, IsEVs, or mPEG-CDM-IsEVs under pH 7.4 and pH 6.0 for 2 h. EdU-positive cells (green); DAPI-stained nuclei (blue). Scale bar: 10 μm. (f) Statistical analysis of EdU-positive cells from (e). (g) Representative tube formation images of bEnd.3 cells treated with PBS, IsEVs, or mPEG-CDM-IsEVs under pH 7.4 and pH 6.0 conditions. Cells were cultured on Matrigel-coated wells for 6 h, and tube-like structures were visualized under a light microscope. Scale bar: 50 μm. (h) Statistical analysis of total tube length from (g). Data are shown as mean ± SD (n = 5). ∗p < 0.05; ∗∗p < 0.01; ∗∗∗p < 0.001; ns, not significant. (For interpretation of the references to colour in this figure legend, the reader is referred to the Web version of this article.)

These findings illustrate the pH-dependent regulation of endothelial cell migration and proliferation by mPEG-CDM-IsEVs. The PEG coating suppresses bioactivity in neutral, non-pathological conditions to minimize off-target effects, while its detachment in acidic environments associated with ischemic stroke restores IsEV functionality. This pH-responsive behavior highlights the potential of mPEG-CDM-IsEVs as a therapeutic platform for targeted vascular regeneration in ischemic strokes.

### 3.6 DOX-loaded PEG-CDM-sEVs for orthotopic glioma inhibition

The therapeutic potential of mPEG-CDM-modified glioma-derived sEVs loaded with DOX was evaluated in an orthotopic GL261 glioma-bearing mouse model. The experimental timeline, encompassing tumor implantation, intravenous treatments, and subsequent analyses, is presented in Fig. 6a. Survival analysis demonstrated that mice treated with mPEG-CDM-GsEVs-DOX exhibited significantly prolonged survival compared to PBS (control), free DOX, or unmodified GsEVs-DOX (Fig. 6b), demonstrating the enhanced therapeutic efficacy achieved through pH-responsive PEG detachment. Fluorescence imaging and MFI analysis of tumor sections confirmed markedly higher DOX accumulation and retention in tumors treated with mPEG-CDM-GsEVs-DOX compared to other groups (Fig. 6c and d). These results underscore the capacity of the pH-responsive PEG coating to achieve precise drug release and reduce off-target effects, thereby improving therapeutic outcomes. Histological evaluations, including H&E staining and tumor volume measurements, revealed a significant reduction in tumor size in the mPEG-CDM-GsEVs-DOX group compared to all other groups (Fig. 6e and f). Furthermore, immunohistochemical staining and Ki-67 quantitative analysis indicated minimal tumor cell proliferation in this group, reflecting its strong anti-tumor activity (Fig. 6g and h). Safety was assessed via H&E staining of major organs (heart, liver, and kidneys). No significant pathological changes were observed in any treatment group, including mPEG-CDM-GsEVs-DOX, confirming the platform's favorable safety profile without evident systemic toxicity (Fig. S11).

**Fig. 6:**
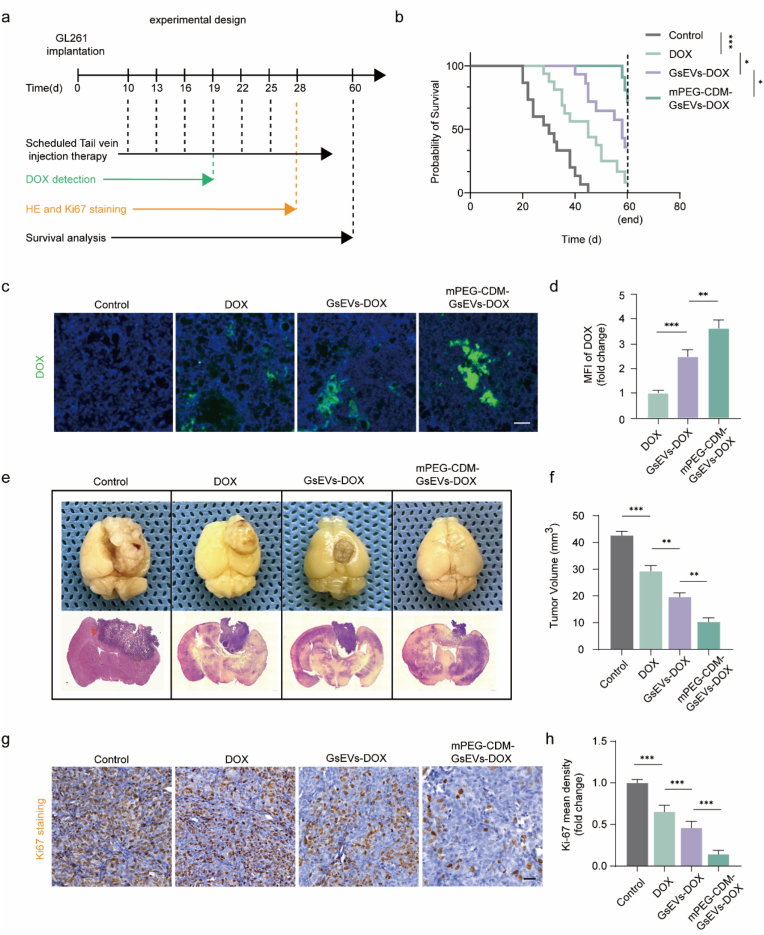
Therapeutic evaluation of mPEG-CDM-GsEVs-DOX in GL261 glioma. (a) Experimental timeline illustrating GL261 cell implantation, DOX treatment regimen, and tissue collection for analysis. (b) Kaplan-Meier survival curves of glioma-bearing mice treated with PBS (control), free DOX, GsEVs-DOX, or mPEG-CDM-GsEVs-DOX (n = 15). (c) Representative fluorescence images of DOX (green) in brain tissues from different treatment groups. Scale bar: 50 μm. (d) Statistical analysis of MFI from (c) (n = 5). (e) Representative gross brain images and HE-stained sections showing tumor burden in each treatment group. (f) Statistical analysis of tumor volume from (e) (n = 5). (g) Representative Ki67-stained brain sections evaluating tumor proliferative activity in different treatment groups. Scale bar: 50 μm. (h) Statistical analysis of Ki67 mean density from (g) (n = 5). Data are shown as mean ± SD. ∗∗p < 0.01; ∗∗∗p < 0.001. (For interpretation of the references to colour in this figure legend, the reader is referred to the Web version of this article.)

In summary, mPEG-CDM-GsEVs-DOX effectively integrates tumor-specific drug delivery with a reliable safety profile, leveraging pH-responsive properties to maximize therapeutic precision and efficacy. Together, these findings establish this platform as a promising strategy for glioma therapy. In this study, we employed a saponin-mediated protocol that simultaneously enabled DOX loading and depletion of endogenous nucleic acids and proteins from GsEVs, as established in our previous work [33]. This approach preserved membrane integrity while reducing the likelihood of cargo-associated tumor promotion. GsEVs were selected based on their intrinsic glioma-homing capability, which supports their application in targeted drug delivery. While these strategies mitigate potential risks, the tumor-derived origin of GsEVs remains a relevant caveat, and further safety evaluations will be essential prior to clinical translation.

### 3.7 pH-responsive mPEG-CDM-IsEVs enhance recovery in ischemic stroke

The therapeutic efficacy of mPEG-CDM-IsEVs in ischemic stroke recovery was assessed using the MCAO reperfusion model, focusing on their effects on infarct volume, vascular repair, and neurological improvement. MAP2-stained brain sections revealed significantly reduced infarct volumes in the mPEG-CDM-IsEV-treated group compared to both IsEV-treated and PBS control groups (Fig. 7a and b). This reduction underscores their ability to alleviate ischemic damage by targeting acidic ischemic regions through the pH-responsive detachment of the PEG coating. Moreover, CD34 and EdU immunofluorescence staining revealed increased vascular density and cellular proliferation in the ischemic brain regions of the mPEG-CDM-IsEV-treated group compared to the other groups (Fig. 7c), underscoring their dual role in restoring vascular networks and stimulating endogenous cell proliferation for tissue recovery. Neurological function was assessed at 0, 1, 2, and 3 days post-reperfusion. Mice treated with mPEG-CDM-IsEVs consistently achieved better neurological scores than those treated with unmodified IsEVs or PBS, reflecting their superior neuroprotective and regenerative effects (Fig. 7d). Motor coordination and sensory responsiveness were evaluated through forelimb grip strength, adhesive touch, and adhesive removal tests (Fig. S12). The mPEG-CDM-IsEV-treated group outperformed the other groups across all tests and time points, demonstrating significant recovery of motor and sensory functions (Fig. 7e). These functional improvements align with the reductions in infarct volume and the enhancements in vascular repair and cellular proliferation observed in this group.

**Fig. 7:**
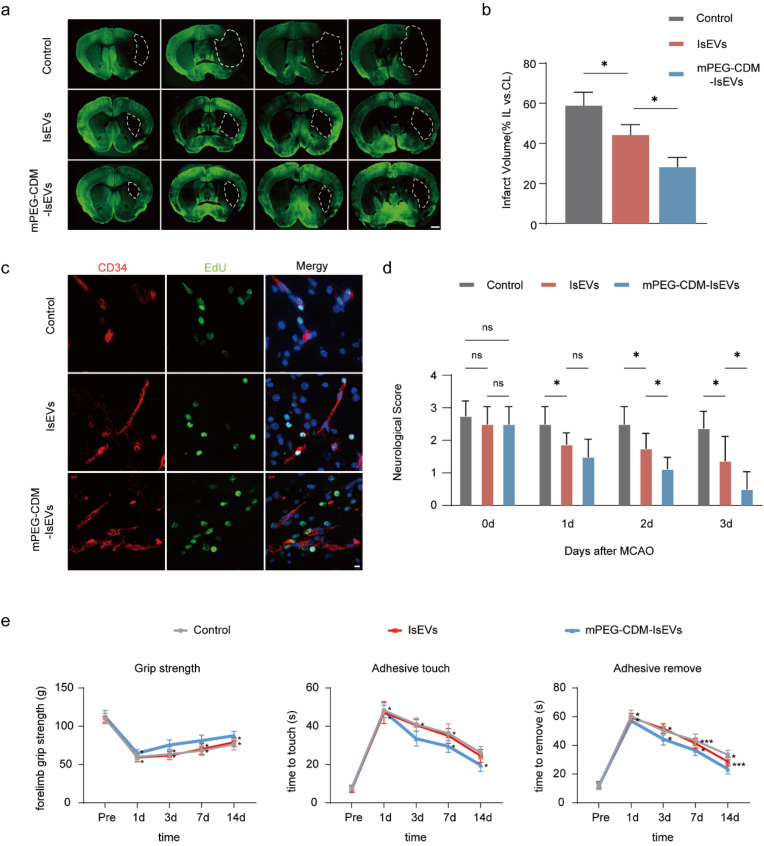
Therapeutic efficacy of mPEG-CDM-IsEVs in MCAO reperfusion. (a) Representative consecutive coronal brain sections showing infarct areas outlined by white dashed lines in PBS (control), IsEVs, and mPEG-CDM-IsEVs groups. Scale bar: 1 mm. (b) Statistical analysis of infarct volume from (a) (IL: ipsilateral side, CL: contralateral side). (c) Representative fluorescence images of brain sections stained for CD34 (red), EdU (green), and DAPI (blue) to assess endothelial and proliferating cells. Scale bar: 10 μm. (d) Neurological scores evaluated daily for 3 days after treatment with PBS (control), IsEVs, or mPEG-CDM-IsEVs. (e) Behavioral assessments including forelimb grip strength, adhesive touch test, and adhesive removal test performed at baseline (Pre-treatment) and at 1, 3, 7, and 14 days post-treatment. Data are shown as mean ± SD (n = 5). ∗p < 0.05; ∗∗p < 0.01; ∗∗∗p < 0.001; ns, not significant. (For interpretation of the references to colour in this figure legend, the reader is referred to the Web version of this article.)

By effectively targeting ischemic microenvironments and minimizing off-target effects, mPEG-CDM-IsEVs provide a robust and precise therapeutic platform for ischemic stroke. Their ability to surpass unmodified IsEVs in both delivery specificity and therapeutic efficacy underscores their potential as an advanced intervention for ischemic stroke treatment.

In comparison to existing EV surface modification strategies—such as lipid insertion, covalent crosslinking, and polymer adsorption—our pH-responsive mPEG-CDM coating offers a unique advantage by reversibly shielding surface ligands under physiological pH and re-exposing them in acidic disease microenvironments. This dynamic feature helps balance systemic stability with targeted delivery. While conventional PEGylation improves circulation time, it often compromises cellular uptake due to permanent surface masking. In contrast, the acid-cleavable mPEG-CDM strategy enables functional restoration of sEVs at pathological sites, particularly relevant for glioma and ischemic stroke where local acidosis facilitates PEG detachment. Moving forward, integrating additional targeting ligands or responsive release elements may further enhance the therapeutic potential of this modular platform for neurological disease treatment.

## 4 Conclusion

In this study, a novel acid-removable PEGylation strategy for sEVs was successfully developed. The acid-labile PEG coating effectively reduced macrophage uptake under physiological conditions while enhancing internalization by target cells in acidic environments. This dual response facilitated the passive enrichment of sEVs at glioma and ischemic stroke sites, thereby improving therapeutic efficacy in both diseases. Overall, this strategy provides valuable insights into the rational design of surface-engineered sEVs as delivery platforms for disease-specific therapy.

## CRediT authorship contribution statement

**Jianwei Zhao:** Writing – original draft, Project administration, Investigation. **Xinyu Niu:** Supervision, Investigation. **Lei Luo:** Validation, Methodology. **Ji Yuan:** Supervision, Methodology. **Juntao Zhang:** Visualization, Methodology. **Xin Niu:** Validation, Data curation. **Hengli Tian:** Resources, Funding acquisition. **Yunlong Yang:** Validation, Methodology, Conceptualization. **Zhifeng Deng:** Writing – review & editing, Funding acquisition, Conceptualization. **Yang Wang:** Validation, Conceptualization.

## Declaration of competing interest

The authors declare that they have no known competing financial interests or personal relationships that could have appeared to influence the work reported in this paper.

## Figures and Tables

**figs1:**
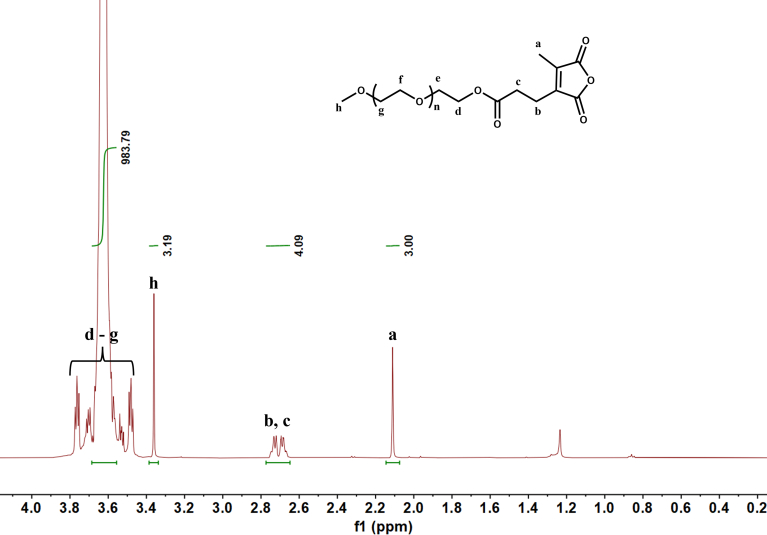


**figs2:**
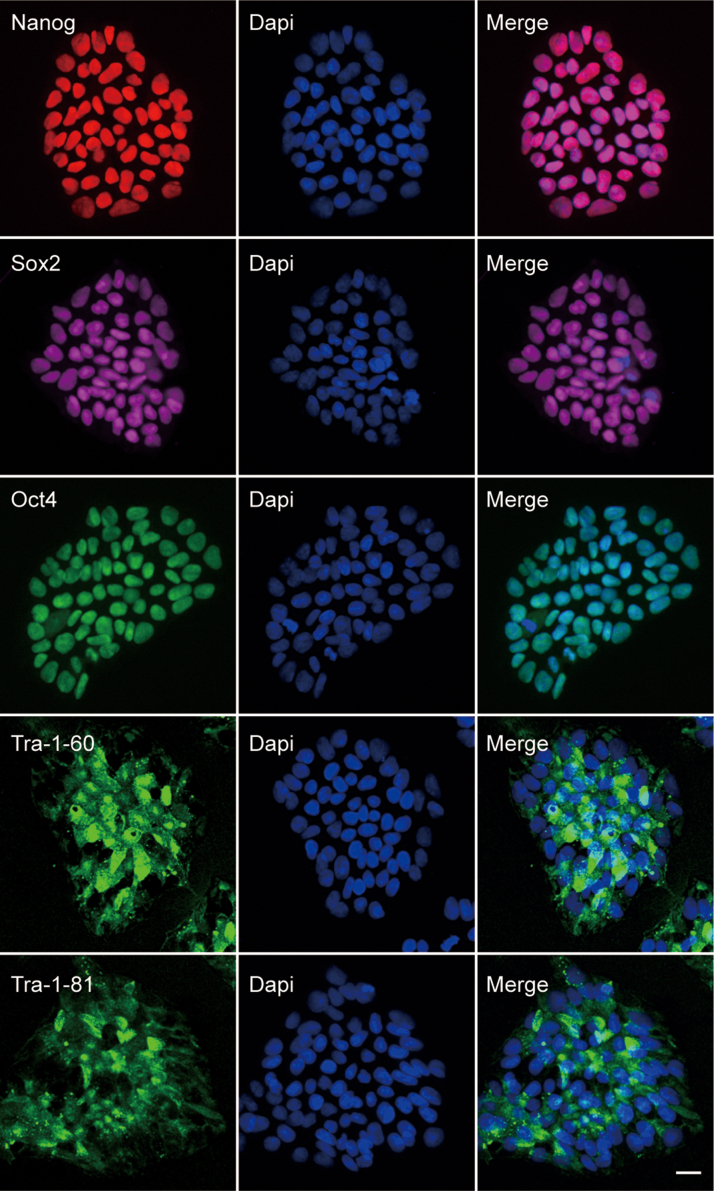


**figs3:**
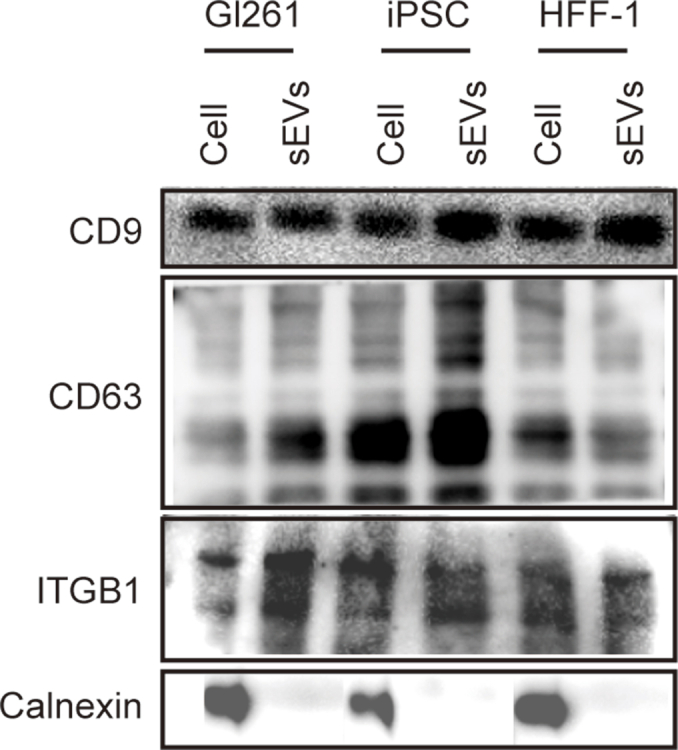


**figs4:**
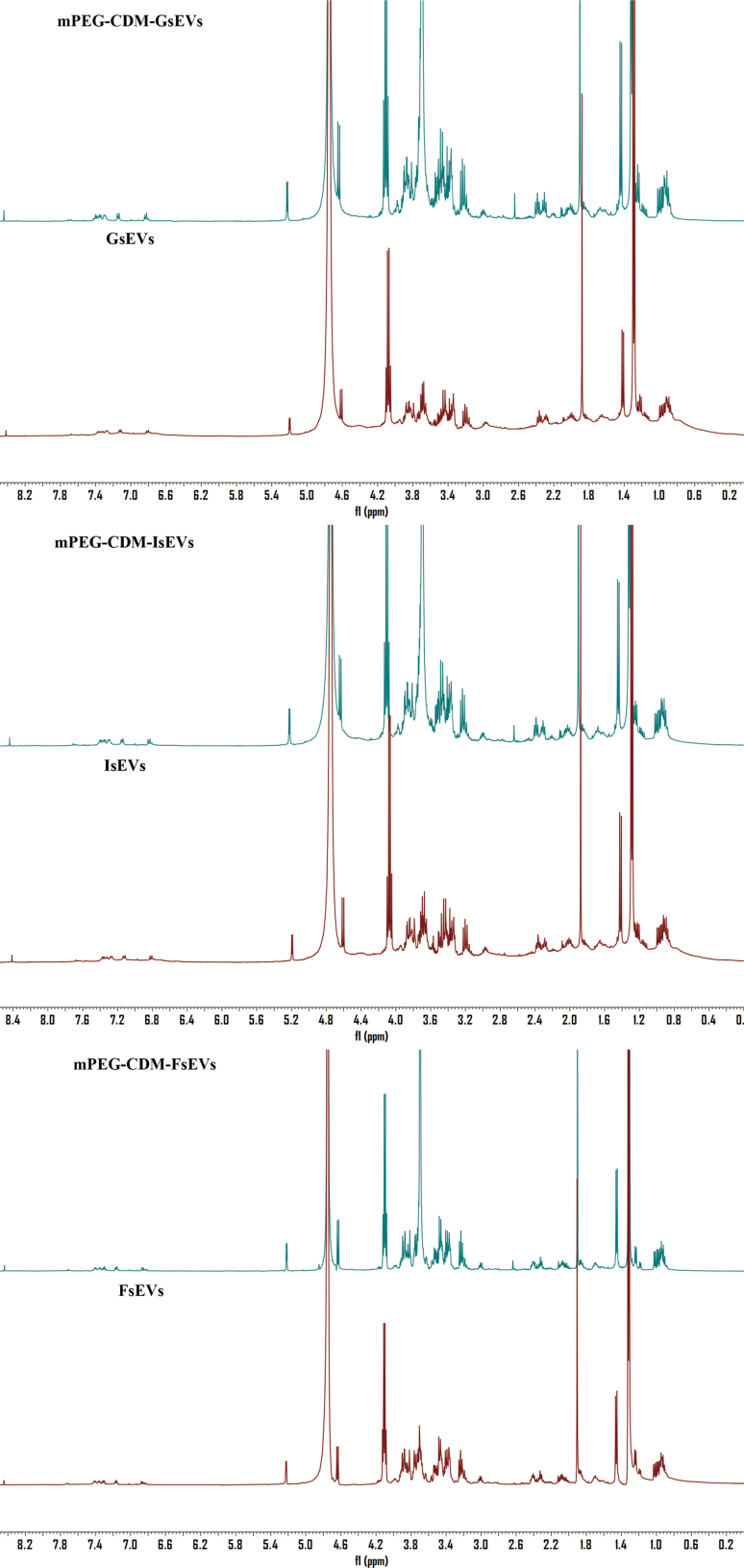


**figs5:**
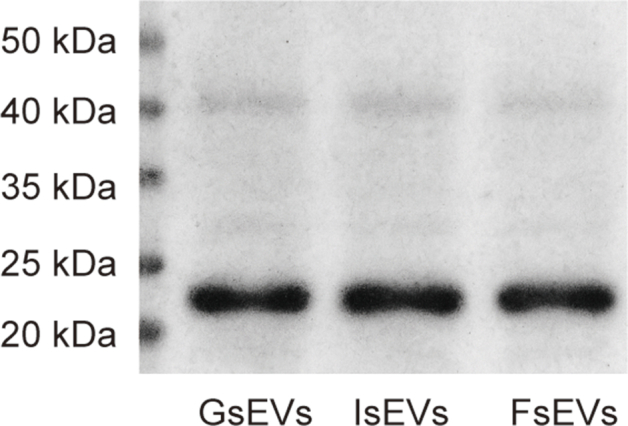


**figs6:**
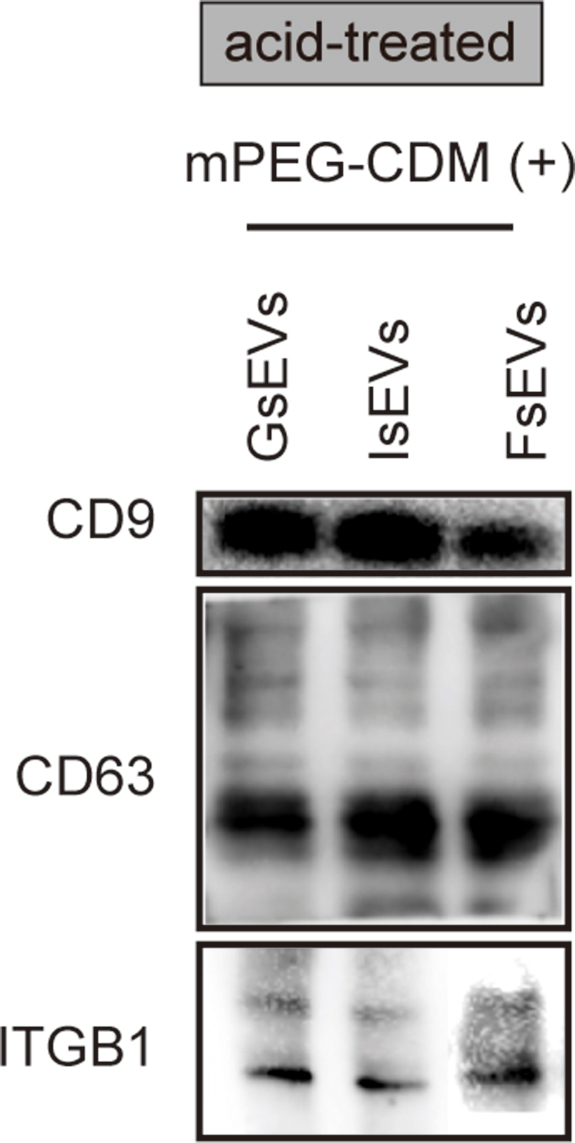


**figs7:**
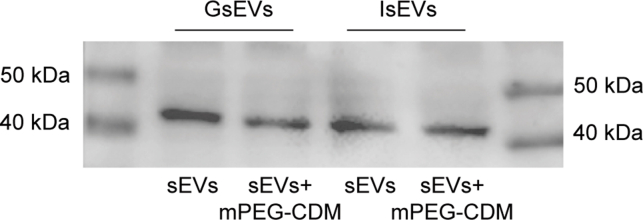


**figs8:**
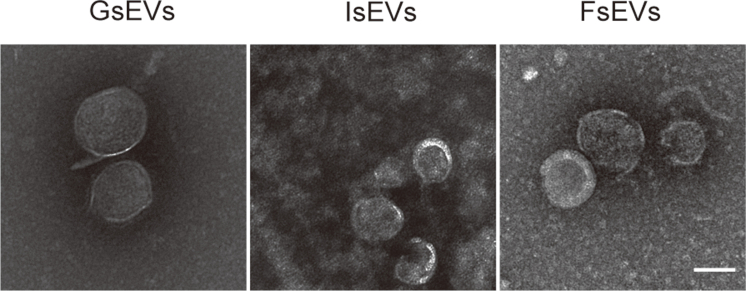


**figs9:**
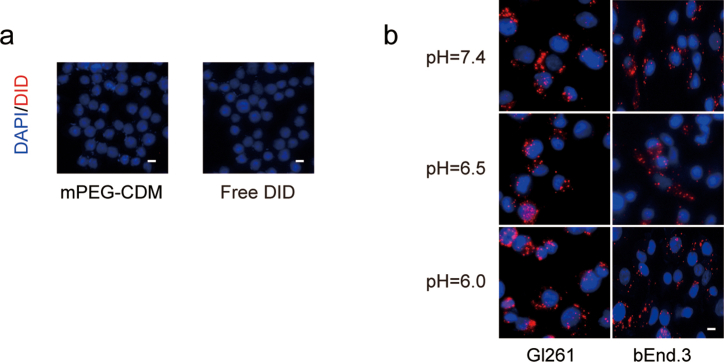


**figs10:**
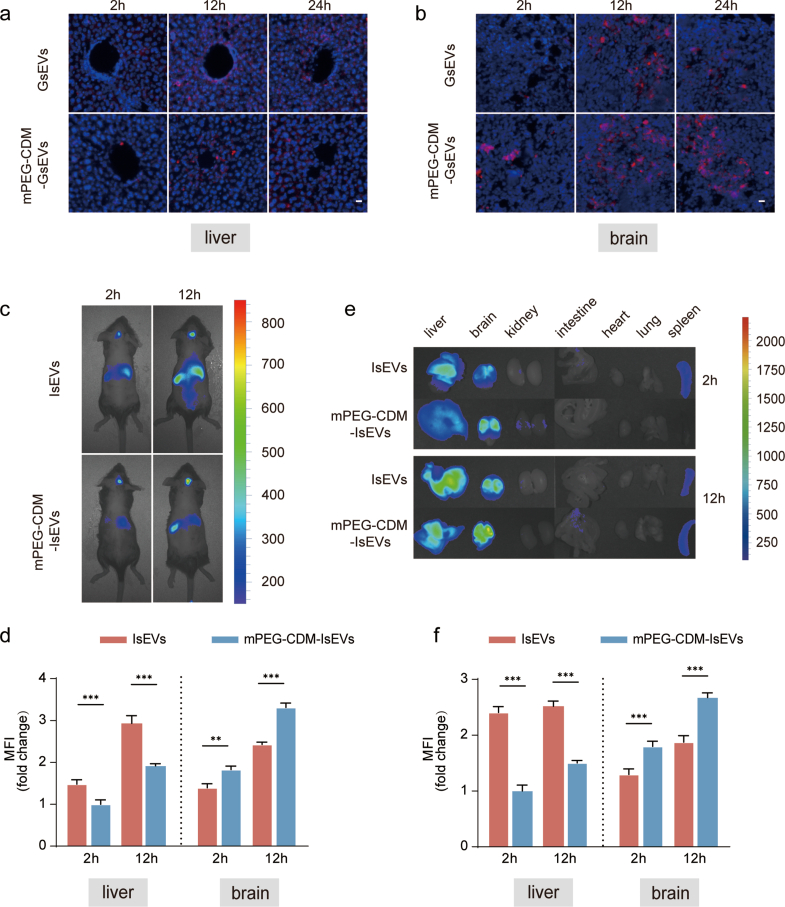


**figs11:**
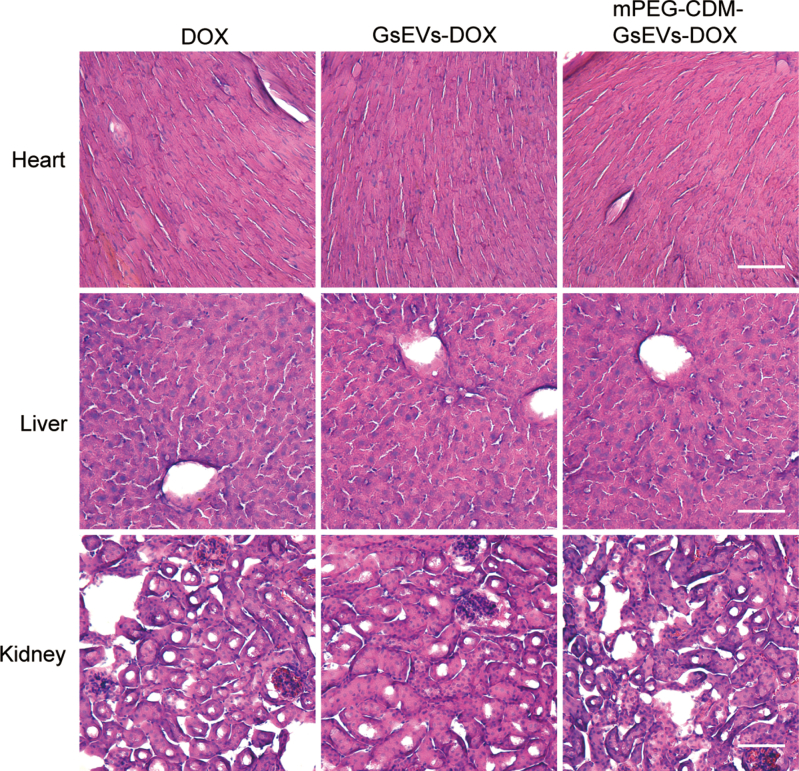


**figs12:**
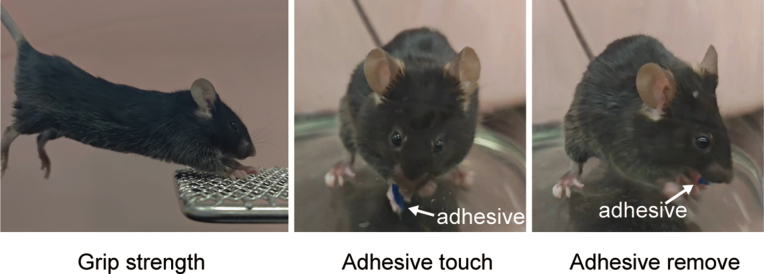


## Data Availability

Data will be made available on request.
